# Connected Communities | Learning lessons from person-centred community-based support services’ implementation: a mixed-methods study protocol.

**DOI:** 10.3310/nihropenres.13494.2

**Published:** 2024-11-12

**Authors:** Danielle L. Christian, Kathryn Berzins, Jo C. Weldon, Madalina Toma, Mark Gabbay, Caroline Watkins, Julien Forder

**Affiliations:** 1 Applied Health Research Hub (AHRh), University of Central Lancashire, Preston, PR1 2HE, UK; 2 NIHR Applied Research Collaboration North West Coast (ARC-NWC), University of Liverpool, Liverpool, L69 3GL, UK; 3 NIHR Applied Research Collaboration (ARC) Kent Surrey and Sussex, Personal Social Service Research Unit (PSSRU), School of Social Policy, Sociology and Social Research, University of Kent, Canterbury, CT2 7NF, UK; 4 Department of Primary Care, University of Liverpool, Liverpool, L69 3GL, UK

**Keywords:** Social prescribing, community-based, implementation, CFIR, NPT, social care, person-centred, social support

## Abstract

**Background:**

Person-centred community-based support services (PCCBSS) are an array of non-clinical services provided by organisations such as NHS Trusts, voluntary sector organisations, or local authorities.

All PCCBSS involve an individual (variously known as a 'social prescriber’, ‘link worker’, ‘signposter’, ‘navigator’, ‘connector’ or ‘neighbourhood coach’) who talks with a service user before directing them to a range of relevant community sources of social, emotional, and practical support.

Despite much recent investment in social prescribing, and its increased prominence within the policy context across England, little is understood about how PCCBSS are implemented. Research is required across different contexts to describe PCCBSS implementation; in particular, how social care providers successfully interact to support the implementation of PCCBSS, and how services responded to circumstances imposed by the COVID-19 pandemic.

**Purpose:**

The aim of this post-implementation mixed-methods study is to explore how PCCBSS are implemented and become part of usual working practice. Using three services in North West England as case studies, we will examine factors influencing PCCBSS implementation and establish where there is learning for the wider adult social care system.

**Focus:**

The study comprises two work packages (WPs):

WP1: collecting data by reviewing service documents from three PCCBSS case studies;

WP2: interviewing staff and service users (≤20 participants per PCCBSS);

Key implementation data will be systematically abstracted (from WPs1&2) into a coding frame; combining contextual determinants from the Consolidated Framework for Implementation Research (CFIR) with process-related domains from Normalization Process Theory (NPT).

**Key outputs:**

The findings from WP1 and WP2 will be presented in the form of an illustrated ‘pen portrait’, developed collaboratively with Applied Research Collaboration North West Coast ARC NWC public advisers, to illustrate how implementation evolved for each of the PCCBSS across key time-points in the process (initiation; operation; maintenance). The findings will also inform an online implementation toolkit providing recommendations for setting up future PCCBSS.

## Plain Language Summary

Person-centred community-based support services (PCCBSS) are services that direct people to a range of activities that might help them improve their health and wellbeing. There is a lack of understanding about how these support services are put into practice, how services work with each other, and how these services responded to the COVID-19 pandemic.

The aim of this study, named ‘Connected Communities’, is to find out how existing PCCBSS have been set up, and to provide recommendations for those hoping to do something similar.

There are two parts to the study: work package 1 (WP1) and work package 2 (WP2). WP1 will review existing documents from three PCCBSS, including published and unpublished reports, and extract any relevant information about how the services were set up. WP2 will interview service providers (staff in the PCCBSS who help support individuals) and linked providers (professionals who work in, or with, the PCCBSS either to refer individuals, or run local community services). These interviews aim to explore how each service compares to others, the experience of working for, or with, the service, and factors that make the service easier or harder to deliver. People who use the service (service users) will also be interviewed to find out what support they received, how well they felt their needs were supported, and their understanding of the service. The information about how the services were set up and delivered will be put into a framework, or selection of implementation factors, looking at the setting, the people involved, the design of the PCCBSS and the process of setting the services up. The findings from this ‘Connected Communities’ study will inform a list of recommendations, a sort of toolkit, for people wanting to set up similar services in the future.

## Introduction

Person-centred, community-based support services (PCCBSS) are defined as a range of non-clinical services that are provided by statutory and third sector organisations ( Featherstone *et al.*, 2022; HM Government Digital Service; NHS England and NHS Improvement, 2020). Community-based support includes an array of personalised activities to support individuals in improving their health and wellbeing. Individuals accessing such support (through professional, or self-, referral) may have a wide range of social, emotional, physical, or practical needs. These services generally involve a ‘signposter’ who supports individuals to identify their own needs before directing them to relevant local sources of support in their community.

There are many forms of PCCBSS operating across communities, with ever-changing terminology and foci ( *e.g.* UK Research and Innovation has recently adopted 'self-driven healthcare' as an umbrella term). PCCBSS present in different models and taxonomies, although the most frequently used term is ‘social prescribing’, which is now (published after completion of our data collection) internationally accepted to be conceptually defined as *“a means for trusted individuals in clinical and community settings to identify that a person has non-medical, health-related social needs and to subsequently connect them to non-clinical supports and services within the community by co-producing a social prescription — a non-medical prescription, to improve health and well-being and to strengthen community connections”* (Muhl, 2023). Social prescribing is generally a concept considered to be situated within health services and local authorities (with some cross-over with voluntary services), though it is unclear how social care providers successfully interact with health services, local authorities, and voluntary services. For the purposes of the ‘Connected Communities’ study, we are interested in any services (not exclusively social prescribing) where an individual is (self-)referred to a PCCBSS that uses a signposter to consult with a service user to identify their needs or support individuals to access other services (regardless of setting, referral route, operating organisation or funding model).

The evidence for the effectiveness of social prescribing indicates an absence of high-quality research (Bickerdike, 2017; Khan, 2024; Napierala, 2022; Pescheny, 2020; Polley, 2022), although studies have reported almost wholly positive impacts from social prescribing (while being limited by a dearth of long-term controlled study availability), with a modest reduction in the use of healthcare resources, and benefits to patients through improvement to their mental and physical health ( Dayson *et al*., 2022; Napierala, 2022; National Academy for Social Prescribing, 2022; Polley, 2022). Social prescribing is gaining traction though, with NHS England investing to create an effective infrastructure for social prescribing in primary care as part of the more personalised care approach of the NHS Long Term Plan ( NHS, 2019). As a result, the aim was that over 1000 trained social prescribing link workers would be in place by the end of 2020/21, enabling at least 900,000 people to be referred to social prescribing by 2023/24 ( NHS, 2019). Despite much recent investment in PCCBSS, further evidence is particularly necessary to describe how these services are implemented ( Dayson *et al*., 2022). Moreover, this implementation took place before, during, and after the COVID-19 pandemic, which must be considered. During this period, link workers reported a decline in referrals to libraries and museums, given the temporary closures of venues or social distancing procedures ( Tierney *et al*., 2022), and the cessation of various community activities due to lockdown restrictions ( Fixsen *et al*., 2022). Additionally, some services moved online, creating issues with technological resources or digital literacy for older populations ( Tierney *et al*., 2022), though increased communication and engagement with others ( Fixsen *et al*., 2022).

Consequently, research is required across different PCCBSS delivery contexts to describe PCCBSS implementation, to examine how contextually-bound they are, and how these services were (un)able to respond to circumstances imposed by the COVID-19 pandemic.

### Main research question/Aims and objectives

The aim of this post-implementation mixed-methods study is to explore the process of embedding a new person-centred community-based support service into usual working practices, and to identify the contextual factors affecting PCCBSS implementation. With regards to ‘Connected Communities’, we are specifically interested in any PCCBSS (across a differentiating range of service intensities, which may (not) include additional support beyond signposting (e.g. models indicated by Husk, 2019)) where a signposter consults with a service user, who is (self-)referred, to identify their needs or support to access other local services (regardless of setting, referral route, operating organisation or funding model).

### Research sub questions

a) What organisational contextual factors affect services' implementation (internal impacts)?

b) How did PCCBSS adapt to delivery and workflow changes imposed by the COVID-19 pandemic (temporal impact)?

## Methods

### Study design

This post-implementation mixed-methods study comprises two work packages (WPs): WP1 involves collecting data by reviewing existing service documents from three PCCBSS case studies (identified by convenience snowball sampling of networked member organisations to NIHR ARC NWC, and representative of a local intersecting network of PCCBSS regionally), and WP2 entails interviewing staff and service users (≤20 participants per PCCBSS).

This study combines two widely-used implementation theoretical tools in a single framework for data collection and analysis to identify and explain key aspects of implementing new interventions, as also undertaken in earlier implementation work ( Burn *et al.*, 2020; Schroeder, 2022).

Key implementation data will be systematically abstracted (from WPs1&2) into a coding frame, combining contextual determinants from the Consolidated Framework for Implementation Research (CFIR) ( Damschroder *et al*., 2009) with process-related domains from Normalization Process Theory (NPT) ( May & Finch, 2009). These final findings will form an online implementation toolkit to guide the development of future PCCBSS.

### WP1: Documentary review of the implementation of PCCBSS services

***Data collection*.** Three case study PCCBSS, from two operating organisations, were identified through their existing relationship with the Applied Research Collaboration North West Coast (ARC NWC). To be eligible, a PCCBSS must have an identifiable operating organisation, and use signposters (variously termed ‘link workers’, ‘social prescribers’, ‘community navigators’, *etc.*) to support professionally referred and/or self-referred service users to access appropriate individually indicated networks, groups, and resources.

The implementation of the three case study PCCBSS will be described using existing documents provided by the services themselves, or identified by the study team, through discussion regarding PCCBSS document outputs and publications. These may include published and unpublished reports, outcome data ( *e.g.* numbers of people referred or diverted from accessing services and pre- and post-intervention wellbeing assessments), health inequalities information, GP referral guides, peer-reviewed literature, and logic models. This documentary review will involve indexing relevant evidence to the PCCBSS’ implementation and ongoing operation and abstracting key implementation data over a period of 14 months (July 2022–September 2023).

***Data analysis*.** The study team will abstract implementation data from the documents provided through the systematic use of a bespoke, operationalised, and iteratively developed coding framework. This framework (described in detail in the ‘Combined CFIR and NPT implementation framework’ section) is based on a modification to the Consolidated Framework for Implementation Research (CFIR) ( Damschroder *et al*., 2009) which incorporates process-related domains from Normalization Process Theory ( May & Finch, 2009). This combined coding framework allows abstraction of implementation data from the case study PCCBSS regarding the intervention characteristics (adaptability, complexity), outer setting (knowledge of service user need and resources), inner setting (infrastructure and culture), characteristics of individuals (individual identification with operating organisation), and process (indicating actions taken to initiate, embed, operate, sustain, and evaluate the PCCBSS, as a result of its implementation).

***Appraisal of PCCBSS implementation components*.** When identifying reporting factors which impede or support implementation of PCCBSS, it is also desirable to quantify the strength of evidence accorded to each implementation component to elicit the impact of different aspects of implementation.

The CFIR has an existing rating tool (offering an ordinal scale of seven categories) to capture the availability, valence, and strength of influences upon implementation ( CFIR Research Team, 2014), which we will apply across our own bespoke combined CFIR and NPT ‘Connected Communities’ coding frame. Availability is captured by use of rating component ‘M’ to indicate missing evidence. Valence is expressed through four rating components to indicate positive or negative influences on implementation: X, 0, +, or -. Strength is indicated by two rating components to demonstrate either weak or strong influences on implementation. A score of 1 means there is some evidence, either positive or negative, that lacks specific detail. Whereas a score of 2 means there is strong evidence indicating a positive or negative affect accompanied with specific detail.

We will systematically apply the defined CFIR ratings to content in individual documents, and across our collated documents for review in the following way ( Table 1, mirroring CFIR rating application from Stanford University School of Medicine guidance, Assefa & McGovern, 2019):

**Table 1.** CFIR rating application of PCCBSS implementation components.

**M**	Missing evidence for construct appraisal
**2-**	Strong evidence impeding implementation (strong barrier)
**1-**	Some evidence impeding implementation (barrier)
**0**	No evidence supporting/impeding implementation
**X**	Mixed sentiment/evidence
**1+**	Some evidence supporting implementation (facilitator)
**2+**	Strong evidence supporting implementation (strong facilitator)

As these are subjective judgements, where the strength of supporting evidence is disputed amongst duplicate coders (10% of documents reviewed) in the research team, these discrepancies in judgement will be determined through a third coder to adjudicate.

This process will identify any implementation domain content gaps from the documentary review which consequently need further investigation during the WP2 interviews.

### WP2: Interviews with PCCBSS service providers and service users

***Data collection*.** WP2 firstly aims to test the conclusions drawn in WP1 and obtain more information regarding the implementation of the PCCBSS that may be missing from the documentary review. Secondly, WP2 aims to explore how the pandemic affected the services, any resultant changes to the existing service, and recommendations to influence how future PCCBSS may be implemented.

Interviews will be carried out with ‘service/linked providers’ (n=10 per PCCBSS) and service users (n=10 per PCCBSS). Based upon the three identified PCCBSS, a sample size of 60 participants is anticipated. These numbers for recruitment are considered appropriate to provide adequate data to answer our research question(s); however, if these interviews do not sufficiently populate gaps in the framework further interviews will be carried out.

Service providers are recognised staff within the case study PCCBSS who act in signposter roles, leadership, or support functions. Linked providers are closely linked professional people who work in or with the PCCBSS ( *e.g.* referrers’ in, those who receive referrals out, GP surgeries, voluntary sector hubs, local community services) to support its operation. Eligible service users are defined as any individual who has been (self-)referred to and received support from a case study PCCBSS.

All interviewees will be sought for recruitment via their PCCBSS, through mailing lists and adverts in service premises. Potential participants will be asked to make direct contact with the research team. Both service/linked providers and service users will be purposively sampled to ensure representativeness according to their underlying characteristics: younger adults (18–35 years), middle-aged adults (36–64 years), older adults (65+ years); either sex (M/F); ethnicity (Asian or Asian British; Black, Black British, Caribbean or African Caribbean; Mixed or multiple ethnic groups; White; Other ethnic group ( HM Government Digital Service (2023)).

Interviewees must be aged above 18 years and have capacity to verbally consent to take part in a research interview (children are beyond the scope for inclusion in this study, as we are exploring intersections with Adult Social Care, for which they would be ineligible to access). Interviews will be conducted in English owing to limited study resources; however, if a potential participant requires translation support and this resource is already available to the referring PCCBSS or participant by other means, their participation will be facilitated (and translated documentation produced via collaboration with provided translation support). Furthermore, to facilitate sampling a diverse population, reasonable adjustments will be made as required to support participation.

PCCBSS service users who are interviewed will receive a GBP 25 gift voucher to demonstrate recognition of their participation. This level of payment is aligned with NIHR’s policy (Version 4.0) on payments to public contributors ( NIHR, 2022). Professionals (from healthcare, adult social care, local authority, and VCF organisations) will not be compensated for their time spent taking part in the study (interviews or focus groups).

Interviews may be conducted by telephone, video-conferencing facility (Microsoft Teams) or face-to-face, depending on the preference of the interviewee, and will be scheduled for an hour. Researchers will be flexible to participants’ needs and undertake interviews at a time and date convenient to them, *e.g.*, outside of working hours if this is preferable. Face-to-face interviews will take place on service premises. Interviews will be conducted between February 2023 to September 2023.

Interviewees will be allocated an anonymised participant ID which will be applied to all data resulting from their interview to maintain their anonymity and encourage open discourse.

Interviews will be conducted with a pre-determined and piloted topic guide which aligns with the study coding frame domains, and will be informed by the existing CFIR Interview Guide Tool and corresponding NPT Toolkit. The service user topic guide will be discussed with a PPI group from a local case study service for acceptability and clarity. The WP2 topic guide questions used in this study with service/linked providers and service users can be found in the ‘Extended data’ section below ( Christian *et al.*, 2023).

***Data analysis*.** Each semi-structured interview will be digitally recorded, transcribed verbatim (through NVivo Transcription, an automated natural language processor using machine-learning technology), and checked for accuracy by the research team against the audio file. While artificial intelligence (AI) will support transcription activities, AI will not be used in any part of our qualitative analysis. Data will be analysed using framework analysis, a primarily deductive approach that in this evaluation will use CFIR and NPT as the framework, as described below. It is a systematic approach that aims to identify, describe, and interpret key patterns within and across cases, but also has the flexibility to incorporate additional inductive codes for any data which does not ‘fit’ within the framework. It has five stages: familiarisation with the data, framework identification, data indexing, charting, and mapping and interpretation. Framework analysis is recognized as a useful approach when multiple researchers are working on an evaluation, and for managing large data sets ( Ritchie & Spencer, 1994). It will allow for analysis to identify common themes within and across PCCBSS without losing detail on individual sites. NVivo (Version 14) will support data management and analysis. Taguette and QualCoder are examples of open-access alternatives to NVivo that can perform equivalent functions. Rigour trustworthiness will be ensured via verification strategies, including a proportion of transcripts (10%) communally coded in group analysis meetings, and discrepancies resolved through discussion at analysis meetings.

### Combined CFIR and NPT implementation framework

***Theoretical underpinnings*.** Use of established implementation theories, models or frameworks can help researchers consider and understand how and why implementation succeeds or fails. There is a choice of theoretical frameworks available to guide implementation questions, which may differ in terms of their theoretical perspective ( *e.g.* psychology or sociology), the implementation level ( *e.g.* the individual, the team, the organisation) and their purpose ( *i.e.* whether they aim to identify drivers of implementation, or whether and to what extent implementation occurred). There may be merit in combining different frameworks where innovations are designed in accordance with multiple theoretical perspectives, target multiple levels of implementation, or pursue multiple purposes ( Schroeder *et al*., 2021).

### Consolidated Framework for Implementation Research (CFIR)

Damschroder’s CFIR ( Damschroder *et al.*, 2009) provides a menu of 26 contextual implementation determinants under five overarching domains: intervention characteristics (8 constructs), outer setting (4 constructs), inner setting (5 constructs [+9 additional subconstructs]), individual characteristics (5 constructs), and process (4 constructs [+4 additional subconstructs]).

Importantly, not all CFIR constructs are relevant to every situation, so researchers or implementers may need to choose what is most relevant to their case. In relation to the implementation of PCCBSS, the CFIR enables a more nuanced account of extra-individual implementation drivers (albeit less detail at individual level) as well as a rather basic examination of more dynamic implementation ‘processes’.

In the context of our ‘Connected Communities’ study, CFIR is ideally placed to identify potential static contextual determinants for implementation (both barriers and facilitators) across four out of the five domains, namely intervention characteristics, inner setting, characteristics of individuals, and outer setting. The fifth CFIR domain ‘process’ will not be utilised in our study.

We will operationalise the four retained CFIR domains in the following manner:

Intervention characteristics: PCCBSS will be defined as delivering either high- or low-service intensity, dependent on their activity configuration of core components (fidelities)/adaptable periphery (flexibilities). This distinction in classification will be determined through discussion by the research team after collection of data from both work packages.

For example, a high-intensity service might require PCCBSS users to complete a defined time-specific duration of received support before being given the opportunity to be trained themselves to deliver PCCBSS activities ( *i.e.* ‘champions’). Whereas a low-intensity service might only require PCCBSS users to receive a single instance of service interaction or use volunteers to signpost service users to support or other groups/services.

The definition distinction is useful in providing clarity for our study findings (given not all PCCBSS services are alike in configuration/operation) and future applicability in practice. It also aligns with available literature that acknowledges the breadth of social prescribing activities being understood to be significantly wider than signposting alone (e.g. Kimberlee, 2015 which delineates social prescribing as four types: Signposting, Light, Medium, and Holistic), which echoes our understanding of social prescribing being a higher-intensity PCCBSS than the simpler signposting provided by lower-intensity services.

Under this domain’s Adaptability construct we will address findings relating to research sub-question b) *How did PCCBSS adapt to delivery and workflow changes imposed by the COVID-19 pandemic (temporal impact)?*

Outer setting: We will capture the identified needs and available resources for service users accessing the PCCBSS, with a focus on differing demographics, degree of isolation and poverty. Factors detailed in PESTLE (Political, Economic, Social, Technological, Legal and Environmental) ( CIPD, 2023) analyses are anticipated to be captured under this domain.

Inner setting: We will consider all types of PCCBSS host sites (by sector, considering both complexity and integration) and those that provide the majority of referrals to, or receive referrals from, carers centres or GP practices.

Under this domain’s constructs we will address findings relating to research sub-question a) *What organisational contextual factors affect services' implementation (internal impacts)?*

Characteristics of individuals involved: We will detail characteristics (attitudes, degree of self-efficacy, and other attributes) of PCCBSS staff, referring services' staff, funder(s), and other linked providers. Where volunteer signposters are used by the PCCBSS to deliver its activities, they will be considered a staff resource under CFIR domain ‘Characteristics of individuals involved’, rather than captured among service user details in CFIR’s domain ‘Outer setting’.

***Normalization Process Theory (NPT)*.** NPT ( May & Finch, 2009) accounts for implementation through analysing the cognitive and social production and organisation of work, the process of establishing practices into routine elements of everyday life, and of sustaining implemented practice into their social contexts.

NPT has four theoretical tenets:

(i) coherence, which supports individual and collective consensus about an intervention and its purpose;

(ii) cognitive participation, the relational work that influences “implementation and legitimation”;

(iii) collective action, the tasks allocated to the various members within the organization to build and sustain use; and,

(iv) reflexive monitoring, the communal appraisal work that aids assessment of the intervention’s introduction.

In the context of this ‘Connected Communities’ study, NPT is used to explore the dynamic processes of how people make sense of, and enact, a new PCCBSS to implement it into usual working practices. We will operationalise the sub-constructs of NPT and substitute these for the excluded CFIR ‘process’ domain, in accordance with CFIR developers’ own guidance ( Damschroder *et al.*, 2009). The implementation processes of each PCCBSS will be detailed according to the four NPT domains (16 sub-constructs), which will be operationalised in the following way for our study:

Coherence: We will describe individual and collective sense-making work among service/linked providers around the PCCBSS.

Cognitive participation: We will detail relational work among service/linked providers which builds and sustains a community of practice for the PCCBSS.

Collective action: We will explore the operational qualities of work done by service/linked providers to enact a set of practices for the PCCBSS.

Reflexive monitoring: We will identify examples of appraisal work to assess and understand how introduction of the PCCBSS affects service/linked providers and service users.

***Application of the combined CFIR and NPT framework in the ‘Connected Communities’ study*.** From the exploration of the above-described frameworks, we saw a clear rationale for combining CFIR with NPT to give more detail to the process elements of implemented PCCBSS.

NPT is key in understanding the process element of implementation which is addressed by CFIR at a rather basic level. This is particularly important as the implementation of social prescribing services requires engagement and collaboration of different individuals, from different organisations, and across different settings.

**Figure 1.**
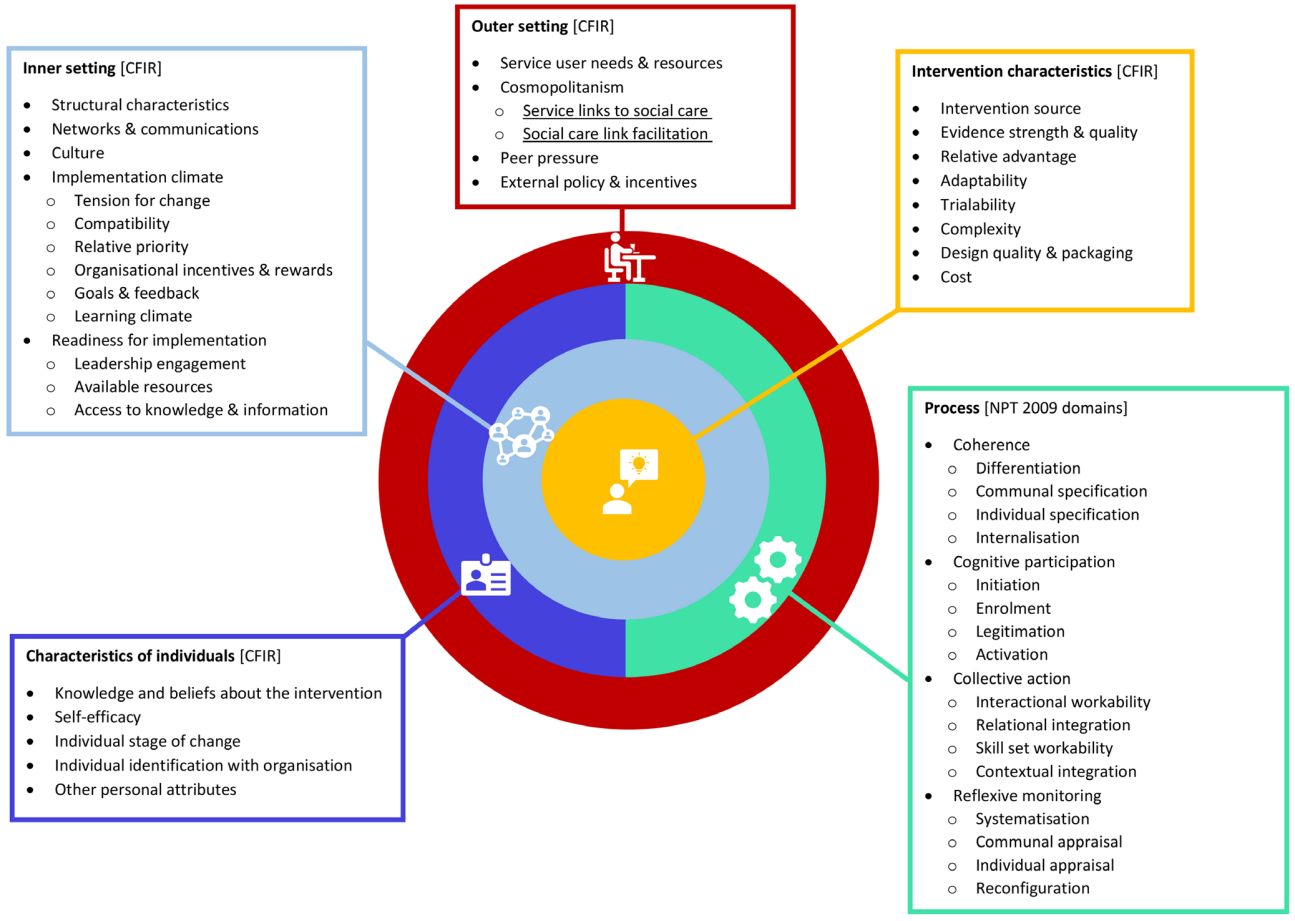
Combined CFIR and NPT implementation coding framework.

Combining CFIR with NPT offers a theoretical lens to illuminate how static contextual factors (identified through CFIR) and dynamic implementation processes (captured by NPT) interact and shape each other ( Schroeder *et al.*, 2021; Schroeder *et al.*, 2022). More specifically, CFIR will be used as an overall framework to guide data collection as it describes qualities of determinants to consider at multiple levels within and beyond the organisation. Figure 1 details the full combined CFIR and NPT implementation coding framework to be used in the ‘Connected Communities’ study.

We will expand an existing defined construct under CFIR’s Outer setting domain (2.B. Cosmopolitanism) with an additional two sub-constructs to accommodate our focused study aim of identifying: PCCBSS service links to social care (2.B.1), and how these social care links are facilitated (2.B.2). For clarity, we note that there are two constructs under CFIR’s 3 ^rd^ domain (Inner setting) which already include additional sub-constructs (3.D. Implementation climate: 6 sub-constructs; 3.E. Readiness for implementation: 3 sub-constructs), and that these are also included within our bespoke coding frame for this study.

Given that NPT constructs give more granularity to the implementation process, we will integrate all four NPT domains (and 16 sub-constructs) as nodes in the combined framework’s process domain, which will provide explanatory strength around why, and how, change occurs to support introducing and sustaining PCCBSS in usual working practices. A full operationalisation of the combined CFIR and NPT implementation coding framework used within this study can be found in the ‘Extended Data’ section below ( Christian *et al.*, 2023).

***Recently updated frameworks*.** We recognise that the team behind the development of NPT 2009 ( May & Finch, 2009) has recently published an updated model which incorporates contextual components affecting implementation as a ‘coding manual’ ( May *et al*., 2022). However, upon comparative scoping by the research team, we reflected that May *et al*., 2022 was better suited for realist evaluation (owing to its modelling of contexts, mechanisms, and outcomes) and that our combined CFIR + NPT 2009 coding frame more explicitly unpicked and permitted appraisal of specific components affecting implementation.

Furthermore, this new coding manual ( May *et al*., 2022) has yet to be used practically by research teams. Consequently, despite the potential to future-proof our work by adopting the updated NPT model (May, 2022), we determined our integrated CFIR and NPT 2009 coding framework was the most appropriate fit for our study.

### Patient and Public Involvement (PPI)

This ‘Connected Communities’ protocol outlines a National Institute of Health and Care Research (NIHR) Applied Research Collaboration (ARC) National Priority study. The National Priorities Programme (NPP) for adult social care and social work is a collaboration between nine ARC partners across England, comprising of public advisers and researchers with expertise in health and social care. The NPP’s public advisory panel selected Topic 9: Using community resources to improve wellbeing, with an average score of 2.77 ( Toma *et al.*, 2021). This topic has subsequently been developed in to ‘ Connected Communities’. The NPP aims to support and stimulate the implementation of evidence-based (evaluated) service change at national and/or supra-regional level in adult social care and social work, as identified by care users and carers, practitioners, professions, researchers, and the wider public ( Toma *et al.*, 2021).

PPI will take place at three levels: national, local, and case study level.

At a national level, this study is being funded through the National Priorities Programme for Adult Social Care and Social work. As such, it will be supported by its wider infrastructure, including its public advisers who are service users and carers as part of the wider National Lived Experience Strategy Group.
We will present aspects of this study at appropriate intervals during the project to ensure consultation, and also capture meaningful views from these key stakeholders about the diverse recruitment materials (especially an easy-read participant information sheet, and participant information video based on ‘easy-read' content) created to support inclusion and gain insights to specific service user experiences of PCCBSS context.
At a local level, PPI will be supported on an ongoing basis by two Public Advisers with experience of community-based support services attached to the project, funded by NIHR Applied Research Collaboration North West Coast.
At a case study service level, PPI will involve consulting with an existing service user advisory group on the topic guides (before interviews are conducted) and the presentation of the emerging findings.

The public were not involved in the design and conduct of the study, the choice of outcome measures, or recruitment to the study, but will be integral in reviewing and refining public-facing materials, engaging in initial coding and analysis interpretation discussions, and designing and agreeing plans for dissemination of the study findings and recommendations for moving this work forward.

### Ethical approval

The ‘Connected Communities’ project has been favourably reviewed by the Camberwell St Giles Research Ethics Committee (IRAS ID: 314796) due to their qualitative project has been favourably reviewed by the Camberwell St Giles Research Ethics Committee (IRAS ID: 314796) due to their qualitative research expertise (date of approval 20/12/2022). All research participants will give verbal informed consent as it is provided immediately before the interview takes place and is considered the definitive, final consent provision (adequacy approved by Ethics Committee). The interviewer will read out each of the statements in the consent form and the participant will have the option to agree or disagree with this statement. All participants are reminded that they have the right to withdraw from the study at any point and verbal consent will only be deemed granted by those participants who agree to participate in the study in accordance with procedures approved by the ethics committee. This study will be conducted in compliance with Health Research Authority (HRA) standards, the study protocol, and Sponsor’s regulatory and monitoring requirements.

## Key outputs and dissemination

### Pen portraits

The analysis from WP1 and WP2 will be presented in the form of an illustrated ‘pen portrait’ to demonstrate how implementation evolved for each of the PCCBSS. Sheard & Marsh (2019) describes the primary purpose of a pen portrait as ‘documenting the journey, story or trajectory of the focus of enquiry in a more or less linear, narrative fashion over the life course of the study’. These pen portraits, modelled on Sheard & Marsh's work (2019) and developed in collaboration with Applied Research Collaboration North West Coast ARC NWC public advisers, will capture key time points in the implementation process for PCCBSS (phases: initiation; operation; maintenance) and provide an overarching engagement profile for each service.

### Online implementation toolkit

Furthermore, we will produce an online implementation toolkit for PCCBSS providing recommendations for practice for those looking to implement similar services in the future. Additional resources will potentially include policy briefs and relevant guidelines for services, peer-reviewed scientific journal articles, accessible reports, lay summaries and conference presentations.

### Dissemination

We will promote knowledge transfer across the wider National Priority Programme for Adult Social Care and Social Work, the ARCs nationally and partner organisations by using all available contacts (within ARC NWC, and across the research consortium led by the University of Kent (ARC KSS)) and the research team’s personal networks. We will maximise this transfer through our established UK (and wider) links, including social media links to the ARC NWC website which will also host the PCCBSS implementation toolkit. We will also seek opportunities to collaborate with the National Academy of Social Prescribing in supporting discussion and dissemination of our resulting PCCBSS implementation toolkit (perhaps via blog, case study or webinar), given social prescribing is covered by its breadth, alongside other lower-intensity person-centred community-based support services.

## Study status

The study has started and data collection for WP1 and WP2 is complete and data analysis is currently underway (as of October 2023).

## Data Availability

No data are associated with this article. OSF: CONNECTED COMMUNITIES | Learning lessons from person-centred community-based support services’ implementation. https://doi.org/10.17605/OSF.IO/TJDP7 ( Christian *et al.*, 2023). This project contains the following extended data: -   Operationalisation of CFIR and NPT domains and constructs for Connected Communities .docx -   Connected Communities WP2 Topic guide V1.2 09 01 2023.docx Data are available under the terms of the Creative Commons Attribution 4.0 International license (CC-BY 4.0).
