# Supplementary material for: Connected Communities | Learning lessons from person-centred community-based support services’ implementation: a mixed-methods study protocol
Source: NIHR Open Res. 2024 Nov 12;3:66. Originally published 2023 Nov 27. [Version 2] doi: 10.3310/nihropenres.13494.2 (PMC11863302; doi:10.3310/nihropenres.13494.2)
Supplement: Peer review history

# Reviewer report: Reviewer response for version 2

**DOI:** 10.3310/nihropenres.14992.r33467

**Published:** 2024-11-22

**Authors:** Michelle Howarth

**Affiliations:** 1 Edge Hill University, Ormskirk, England, UK

The authors response to the original review is robust and well evidenced. They have addressed and/or clarified any areas of ambiguity and strengthened their paper. I look forward to reading the results of this study in the future as the potential impact is significant on policy makers and practice seeking to implement and support PCCBSS>

Is the study design appropriate for the research question?

Yes

Is the rationale for, and objectives of, the study clearly described?

Partly

Are sufficient details of the methods provided to allow replication by others?

Yes

Are the datasets clearly presented in a useable and accessible format?

Not applicable

Reviewer Expertise:

Social prescribing, person centred approaches, grounded theory,

I confirm that I have read this submission and believe that I have an appropriate level of expertise to confirm that it is of an acceptable scientific standard.

# Reviewer report: Reviewer response for version 1

**DOI:** 10.3310/nihropenres.14643.r31074

**Published:** 2024-03-12

**Authors:** Michelle Howarth

**Affiliations:** 1 Edge Hill University, Ormskirk, England, UK

**Overall comments:**

 This is a well constructed and timely protocol. The findings from the study could have meaningful impact on service delivery and development. The data collection methods and analysis proposed are robust and embed PPI views.

 I have made some detailed comments below and indicated potential sources/references for the authors to consider that may strengthen the context and impact of this work. 

 Some of these comments and suggestions relate to the rationale and terminology such as 'signposter' which has been used to describe a range of roles. The authors need to consider how the use of this term could create ambiguity for the reader/user as a result of the challenges influenced through the person centred paradigm that underpins service design. This is integral to the success of PCCBSS and I feel that more consideration of the influence and impact should be included in the rationale - and also in the implementation/dissemination. 

**Rationale & Background:**

 The rationale is strong and this would be a very timely piece of work, however, the background and person centred philosophy needs greater detail.

 The PCCBSS philosophy is predicated on a wellbeing conversation that uses a strengths-based approach to understand 'what matters' to someone, rather than focusing on 'what's the matter with them'. This is a fundamental position which I feel is not clear in the rationale. For example, the authors need to highlight how PCCBSS embraces the person centred through the wellbeing conversation. The authors have described this significant process as occurring when someone  who *" **talks with **a service user before directing them to a range of relevant community sources of social, emotional, and practical support."*. Talking with someone, doesn't really highlight the power or impact of the wellbeing conversation - or the relationship that is formed over a period of time to ensure that the referral is person centred. This is significant because the individualised nature of the conversation and subsequent referral and service provided is underpinned by the person centred philosophy, which, by its very nature, can lead to the use of a range of metrics to capture impact. 

 Those who 'signpost' individual do not typically hold a wellbeing conversation so it is not clear why the term the 'signposter has been used'. The authors may need to consider the different models of social prescribing that as published by Kimberlee (see links below) as the PCCBSS should ideally align with the 'holistic' model which is different from 'signposting'. The use of the term 'signposter' could cause ambiguity as signposting is incongruous with the PCC element.

 Muhl *et al.* (2023 ^1^) global conceptual definition of SP may help to provide a lends on the wider complexities associated with SP that the authors could use (see links below).

https://uwe-repository.worktribe.com/output/927254

https://journals.scholarpublishing.org/index.php/assrj/article/view/808

**Methods:**

 The authors propose to use mixed methods within 2 WP's. This will provide a good helicopter perspective and rich insight into the systems and experiences of implementation of SP. I do have a couple of queries about other data sources that maybe helpful. 

 For example, will the authors also use data from the SP systems which could provide real time and historical data related to the implementation processes - particularly in relation to capturing outcome metrics for commissioning purposes. Will the authors also include referrals that originate from other health professionals and non-health referrers? This maybe particularly relevant if the PCCBSS targets children and young people. 

 The authors have stated that they will use the PPI group to discuss the topic guide - this is reassuring as it will ensure that the data captured are meaningful. Will there also be an opportunity through the PPI events to capture key stakeholders views about 'meaningful' approaches to data collection to ensure that significant contextual data isn't missed?

**Analysis:**

 The data analysis strategy is robust and has integrated validated theoretical frameworks to support the analysis. Can the authors clarify whether any AI will be used as part of the qualitative analysis? 

**Key Outputs & Dissemination:**

 The outputs strategy is good - but I wonder how the online implementation toolkit will be aligned with the National Academy of Social Prescribing (NASP) and rapid evidence reviews - is there an opportunity to collaborate with NASP to support dissemination?

Is the study design appropriate for the research question?

Yes

Is the rationale for, and objectives of, the study clearly described?

Partly

Are sufficient details of the methods provided to allow replication by others?

Yes

Are the datasets clearly presented in a useable and accessible format?

Not applicable

Reviewer Expertise:

Social prescribing, person centred approaches, grounded theory,

I confirm that I have read this submission and believe that I have an appropriate level of expertise to confirm that it is of an acceptable scientific standard, however I have significant reservations, as outlined above.

## References

[1] : Establishing Internationally Accepted Conceptual and Operational Definitions of Social Prescribing Through Expert Consensus: A Delphi Study Protocol. *Int J Integr Care* .2023;23(1) : 10.5334/ijic.6984 3 10.5334/ijic.6984 36741971 PMC9881447

[2] : What is social prescribing?. *Advances in Social Sciences Research Journal* .2015;2(1) : 10.14738/assrj.21.808 10.14738/assrj.21.808

# Response

**Published:** 2024-11-05

**Authors:** Danielle Christian

**Affiliations:** University of Central Lancashire, Preston, England, UK

Dear Professor Michelle Howarth,

 Thank you for taking the time to review our article.

 We really appreciate your expertise and recommendations for improvement to the manuscript. We have responded to each of your suggested amendments below.

 Yours sincerely,

 Dr Danielle Christian, on behalf of the authors

**Reviewer** – The background and person centred philosophy needs greater detail.

 The PCCBSS philosophy is predicated on a wellbeing conversation that uses a strengths-based approach to understand 'what matters' to someone, rather than focusing on 'what's the matter with them'. This is a fundamental position which I feel is not clear in the rationale. For example, the authors need to highlight how PCCBSS embraces the person centred through the wellbeing conversation. The authors have described this significant process as occurring when someone  who "talks with a service user before directing them to a range of relevant community sources of social, emotional, and practical support.". Talking with someone, doesn't really highlight the power or impact of the wellbeing conversation - or the relationship that is formed over a period of time to ensure that the referral is person centred. This is significant because the individualised nature of the conversation and subsequent referral and service provided is underpinned by the person centred philosophy, which, by its very nature, can lead to the use of a range of metrics to capture impact.

 Muhl et al. (20231) global conceptual definition of SP may help to provide a lends on the wider complexities associated with SP that the authors could use (see links below).

*https://uwe-repository.worktribe.com/output/927254*

*https://journals.scholarpublishing.org/index.php/assrj/article/view/808*

 Those who 'signpost' individual do not typically hold a wellbeing conversation so it is not clear why the term the 'signposter has been used'. The authors may need to consider the different models of social prescribing that as published by Kimberlee (see links below) as the PCCBSS should ideally align with the 'holistic' model which is different from 'signposting'. The use of the term 'signposter' could cause ambiguity as signposting is incongruous with the PCC element.

 Kimberlee R: What is social prescribing?. Advances in Social Sciences Research Journal. 2015; 2 (1). 

***Author response*** -We appreciate the reviewer’s concern, but feel this is another instance where there has been confusion between what people understand to be social prescribing, and the breadth of person-centred community-based support services that our project covers (and is inclusive of social prescribing).

 While we acknowledge the wellbeing conversations offer a crucial lens for social prescribers to support their service users, lower-intensity PCCBSS don’t necessarily have this requirement (being simply that a ‘signposter’ role speaks with an individual to establish the support they wish to identify and engage with in their local community – happening superficially with minimal engagement in some cases).

 As we are interested in broader service delivery models than social prescribing, it would be untrue for us to state that every ‘signposter’ conducts wellbeing conversations, despite this happening in higher intensity services. Consequently, we had been deliberately superficial in our descriptor in order to be inclusive. We’re not denying that some PCCBSS have this interaction, but not all do. We wish to highlight also that the suggested conceptual definition reference (Muhl 2023) has replaced our previous (NHS) definition in the Introduction section, and added to the references.

 We were grateful for you drawing our attention to Kimberlee 2015, as it provides very helpful information which indicates that signposting is one aspect (of 4) that social prescribers engage in: " *Based on analysis of local practice this article delineates social prescribing interventions into four types: Signposting, Light, Medium and Holistic."* This is useful for us to reference and refute arguments that using the ‘signposter’ aspect to define PCCBSS is too basic and prevents application to the breadth of role that social prescribers are engaged in. Signposting is part of their engagement, but as Kimberlee 2015 considers that social prescribing interventions can be holistic, it definitely meets our understanding of person-centred support.

 Considering that the breadth of social prescribing activities is understood to be significantly wider than signposting alone echoes our understanding of social prescribing being a higher-intensity PCCBSS than the simpler signposting provided by lower-intensity services.

 We have made this clearer in the text under CFIR Intervention characteristics: *“The definition distinction is useful in providing clarity for our study findings (given not all PCCBSS services are alike in configuration/operation) and future applicability in practice. It also aligns with available literature that acknowledges the breadth of social prescribing activities being understood to be significantly wider than signposting alone (e.g. Kimberlee 2015 which delineates social prescribing as four types: Signposting, Light, Medium, and Holistic), which echoes our understanding of social prescribing being a higher-intensity PCCBSS than the simpler signposting provided by lower-intensity services.”*

**Reviewer** – Will the authors also use data from the SP systems which could provide real time and historical data related to the implementation processes - particularly in relation to capturing outcome metrics for commissioning purposes. Will the authors also include referrals that originate from other health professionals and non-health referrers? This maybe particularly relevant if the PCCBSS targets children and young people.

***Author response*** -We have explicitly stated that we use existing data provided by services, and that this may include outcome data: *“The implementation of the three case study PCCBSS will be described using existing documents provided by the services themselves, or identified by the study team, through discussion regarding PCCBSS document outputs and publications. These may include published and unpublished reports, outcome data (e.g. numbers of people referred or diverted from accessing services and pre- and post-intervention wellbeing assessments), health inequalities information, GP referral guides, peer-reviewed literature, and logic models.”*

 We had implied that we are exploring adult use of PCCBSS alone, but appreciate that this may be more helpful to make explicit: [currently, p6 under WP2 methods] *"Both service/linked providers and service users will be purposively sampled to ensure representativeness according to their underlying characteristics: younger adults (18–35 years), middle-aged adults (36–64 years), older adults (65+ years)"*

*"Interviewees must be aged above 18 years and have capacity to verbally consent to take part in a research interview."*

 Immediately following this sentence, we have now added the following text to be more explicit: *“(children are beyond the scope for inclusion in this study, as we are exploring intersections with Adult Social Care, for which they would be ineligible to access)”*

**Reviewer** – The authors have stated that they will use the PPI group to discuss the topic guide - this is reassuring as it will ensure that the data captured are meaningful. Will there also be an opportunity through the PPI events to capture key stakeholders views about 'meaningful' approaches to data collection to ensure that significant contextual data isn't missed?

***Author response*** - Thank you for this query, unfortunately it is not possible for us to establish other meaningful approaches to data collection owing to use of our framework and methods pre-determining how this would happen (what we would ask and how we would collect data); however, we have  indicated the diversity of recruitment materials to support inclusion and gain insights to specific SU experiences of PCCBSS context, and included under the ‘national level’ PPI section, the following content:

*“We will present aspects of this study at appropriate intervals during the project to ensure consultation, and also capture meaningful views from these key stakeholders about the diverse recruitment materials (especially an easy-read participant information sheet, and participant information video based on ‘easy-read' content) created to support inclusion and gain insights to specific service user experiences of PCCBSS context.”*

**Reviewer** – Can the authors clarify whether any AI will be used as part of the qualitative analysis?

***Author response*** - Great question, and no we have not utilised AI in our qualitative analyses, but it has supported our transcription production. We have made this explicit on p6 under *“Each semi-structured interview will be digitally recorded, transcribed verbatim (through NVivo Transcription, an automated natural language processor using machine-learning technology), and checked for accuracy by the research team against the audio file. While artificial intelligence (AI) will support transcription activities, AI will not be used in any part of our qualitative analysis. Data will be analysed using framework analysis, a primarily deductive approach that in this evaluation will use CFIR and NPT as the framework, as described below.”*

**Reviewer** – The outputs strategy is good - but I wonder how the online implementation toolkit will be aligned with the National Academy of Social Prescribing (NASP) and rapid evidence reviews - is there an opportunity to collaborate with NASP to support dissemination?

***Author response*** - Thank you for this query. Upon reflection and exploration of this comment, we would highlight that we wish to avoid competing/conflicting with or looking to supersede NASP’s own online toolkit which is specifically for social prescribing ((https://socialprescribingacademy.org.uk/resources/social-prescribing-self-assessment-development-guide/#msdynttrid=CQAgyYCDN_X1WqWtUzzaTRfQQXtw4dDGldj1Tgu4_sM). To clarify, ours is a broader focus for different types of person-centred community-based support service, and ours contains no self-assessment functions (as the NASP toolkit does), instead being a static resource for consideration of implementation barriers to avoid and implementation facilitators that can be leveraged when establishing future PCCBSS in communities.

 Further to this, among NASP’s online resources they host blogs, case studies and webinars, and we are happy to make contact to ask if we can share our findings with them in one of those means as a basis for discussion and dissemination. We intend to do this via our host institution’s own Social Prescribing Unit’s lead (Mental Health Research and Knowledge Exchange Lead at UCLan) who is closely linked with NASP already. We have addressed this query in the text, in the following manner:

*“We will also seek opportunities to collaborate with the National Academy of Social Prescribing in supporting discussion and dissemination of our resulting PCCBSS implementation toolkit (perhaps via blog, case study or webinar), given social prescribing is covered by its breadth, alongside other lower-intensity person-centred community-based support services.”*

# Reviewer report: Reviewer response for version 1

**DOI:** 10.3310/nihropenres.14643.r31075

**Published:** 2024-03-12

**Authors:** Hendrik Napierala

**Affiliations:** 1 Charité - Universitätsmedizin Berlin, Berlin, Germany

This is a study protocol for a post-implementation mixed-methods study to assess which contextual factors affect implementation of person-centred community-based support services (PCCBSS) and how PCCBSS adapted to changes during the COVID-19 pandemic.

 The protocol is well written and clearly structured to support transparent reporting of most of the relevant aspects of the project. I think that the manuscript is suitable for indexing. However, it needs some clarifications.

 Here are some comments that hopefully help you in refining the manuscript:

1. I would prefer if the title contains the term study protocol. I do not know if the current title reflects journal standards but it would be easier to distinguish if e.g., found in a database.
2. Is PCCBSS a common term or is it exclusively used in your context? The CUSP framework (1) for Social Prescribing does not necessarily involve someone from the healthcare sector. However, by introducing a new acronym you make it more complex for the reader to distinguish it with "other" concepts (e.g. SP). In my opinion they are both the same and you rather should state how you define Social Prescribing.
3. When citing evidence for the effectiveness you should think about adding more relevant citations. There is a large body of current evidence (e.g. (2-4)). You should also cite relevant projects (5) or evidence (e.g. 6) related to the evidence you want to gather. Currently, the manuscript does not adequately reflect the body of literature.
4. I do not understand how you want to answer question b). Currently, Figure 1 and the explanations for CFIR and NPT do not directly refer to workflow changes. Shouldn't that be part of the outer setting, for example? Please make it clearer in the protocol. I found no further mentions of the topic in the methods section.
5. I would rather suggest to move the main research questions to the end of the introduction and then start with the study design in the methods section. I would move the PPI and ethical approval to the end.
6. Can you provide the interview guide as supplementary material? This would make it easier to "replicate" your methods and use them in other settings.
7. The protocol was published on OSF after the data collection had ended. Please provide a statement on differences between the primary research proposal and the current protocol. Or state that there were none.
8. I am missing information on funding and potential conflicts of interest.

Is the study design appropriate for the research question?

Partly

Is the rationale for, and objectives of, the study clearly described?

Partly

Are sufficient details of the methods provided to allow replication by others?

Partly

Are the datasets clearly presented in a useable and accessible format?

Not applicable

Reviewer Expertise:

Social Prescribing, Mixed-methods evaluations, pragmatic trials, implementation science

I confirm that I have read this submission and believe that I have an appropriate level of expertise to confirm that it is of an acceptable scientific standard, however I have significant reservations, as outlined above.

## References

[1] : Social Prescribing: Systematic Review of the Effectiveness of Psychosocial Community Referral Interventions in Primary Care. *Int J Integr Care* .2022;22(3) : 10.5334/ijic.6472 11 10.5334/ijic.6472 36060831 PMC9389950

[2] : What approaches to social prescribing work, for whom, and in what circumstances? A realist review. *Health & Social Care in the Community* .2020;28(2) : 10.1111/hsc.12839 309-324 10.1111/hsc.12839 PMC702777031502314

[3] : Establishing internationally accepted conceptual and operational definitions of social prescribing through expert consensus: a Delphi study. *BMJ Open* .2023;13(7) : 10.1136/bmjopen-2022-070184 10.1136/bmjopen-2022-070184 PMC1035128537451718

[4] : Social prescribing: less rhetoric and more reality. A systematic review of the evidence. *BMJ Open* .2017;7(4) : 10.1136/bmjopen-2016-013384 e013384 10.1136/bmjopen-2016-013384 28389486 PMC5558801

[5] : Factors influencing social prescribing initiatives: a systematic review of qualitative evidence. *Perspect Public Health* .2023; 10.1177/17579139231184809 17579139231184809 10.1177/17579139231184809 37497769

[6] : Implementing social prescribing in primary care in areas of high socioeconomic deprivation: process evaluation of the 'Deep End' community Links Worker Programme. *Br J Gen Pract* .2021;71(713) : 10.3399/BJGP.2020.1153 e912-e920 10.3399/BJGP.2020.1153 34019479 PMC8463130

# Response

**Published:** 2024-11-05

**Authors:** Danielle Christian

**Affiliations:** University of Central Lancashire, Preston, England, UK

Dear Dr Hendrik Napierala,

 Thank you for taking the time to review our article.

 We have responded to each of your suggested amendments below and really appreciate your expertise and recommendations for improvement.

 Reviewer - I would prefer if the title contains the term study protocol. I do not know if the current title reflects journal standards but it would be easier to distinguish if e.g., found in a database.

- Author response - Great suggestion, completely agree, and now inserted: “Connected Communities | Learning lessons from person-centred community-based support services’ implementation: a mixed-methods study protocol”. Thank you.

Reviewer - Is PCCBSS a common term or is it exclusively used in your context? The CUSP framework (Napierala et al., 2022) for Social Prescribing does not necessarily involve someone from the healthcare sector. However, by introducing a new acronym you make it more complex for the reader to distinguish it with "other" concepts (e.g. SP). In my opinion they are both the same and you rather should state how you define Social Prescribing.

1. Napierala H, Krüger K, Kuschick D, Heintze C, et al.: Social Prescribing: Systematic Review of the Effectiveness of Psychosocial Community Referral Interventions in Primary Care.Int J Integr Care. 2022; 22 (3): 11

- Author response - You have not been alone in assuming that PCCBSS is being used as a byword for social prescribing, as Reviewer 1 also made a similar observation. When we commenced this work we established that we were interested in wider services than social prescribing alone, but needed to find an accurate and explicit descriptor that covered the types of services we were interested in; however we were unable to identify anything inclusive enough that already seemed to be in use, which is where the use of ‘person-centred community-based support services’ began to be utilised for our project. As this quite verbose, after initial explanation in individual documents, we then subsequently refer to it as PCCBSS. As this work is focused on describing PCCBSS (which are broader than social prescribing alone) to cover the breadth of available social interventions that employ the use of a signposter to connect individuals with appropriate local resources, we have consequently addressed this by making our text explicit that we’re being inclusive of different referral routes, different host/operating organisations, different funding models.We have clarified this in the text as follows:Introduction – “For the purposes of the ‘Connected Communities’ study, we are interested in any services (not exclusively social prescribing) where an individual is (self-)referred to a PCCBSS that uses a signposter to consult with a service user to identify their needs or support individuals to access other services (regardless of setting, referral route, operating organisation or funding model).” Main research question/Aims and objectives - "With regards to ‘Connected Communities’, we are specifically interested in any PCCBSS (across a differentiating range of service intensities, which may (not) include additional support beyond signposting (e.g. models indicated by Husk 2019)) where a signposter consults with a service user, who is (self-) referred, to identify their needs or support to access other local services (regardless of setting, referral route, operating organisation or funding model).“

Reviewer - When citing evidence for the effectiveness you should think about adding more relevant citations. There is a large body of current evidence (e.g. (2-4)). You should also cite relevant projects (5) or evidence (e.g. 6) related to the evidence you want to gather. Currently, the manuscript does not adequately reflect the body of literature.

 2. Husk K, Blockley K, Lovell R, Bethel A, et al.: What approaches to social prescribing work, for whom, and in what circumstances? A realist review. Health & Social Care in the Community. 2020; 28 (2): 309-324 

 3. Muhl C, Mulligan K, Bayoumi I, Ashcroft R, et al.: Establishing internationally accepted conceptual and operational definitions of social prescribing through expert consensus: a Delphi study. BMJ Open. 2023; 13 (7). Publisher Full Text

 4. Bickerdike L, Booth A, Wilson PM, Farley K, et al.: Social prescribing: less rhetoric and more reality. A systematic review of the evidence.BMJ Open. 2017; 7 (4): e013384

 5. Ebrahimoghli R, Pezeshki MZ, Farajzadeh P, Arab-Zozani M, et al.: Factors influencing social prescribing initiatives: a systematic review of qualitative evidence.Perspect Public Health. 2023. 17579139231184809 

 6. Chng NR, Hawkins K, Fitzpatrick B, O'Donnell CA, et al.: Implementing social prescribing in primary care in areas of high socioeconomic deprivation: process evaluation of the 'Deep End' community Links Worker Programme.Br J Gen Pract. 2021; 71 (713): e912-e920

- Author response - Excellent suggestions, thank you for indicating their inclusion.

We have selectively incorporated additional available evidence in our updated protocol (although not all, given our project has a broader focus than social prescribing alone and we are wary of giving this more recognised form of PCCBSS significant ‘air time’ and confusing readers to believe the work is solely another euphemism for social prescribing).

 The following publications are consequently now also included in our references:

 Husk 2020, Muhl 2023, Napierala 2022, Bickerdike 2017, Khan 2024, Pescheny 2020 and Polley 2022

 Reviewer – I do not understand how you want to answer question b). Currently, Figure 1 and the explanations for CFIR and NPT do not directly refer to workflow changes. Shouldn't that be part of the outer setting, for example? Please make it clearer in the protocol. I found no further mentions of the topic in the methods section.

- Author response - Thank you for also drawing our attention to this omission.The operationalisation (in Extended data) of our CFIR/NPT framework constructs for 1.d. Adaptability have captured ‘flexibilities/adaptable peripheries’ which are the aspects of how PCCBSS addressed impacts of the pandemic.Our research sub-questions relate to internal (Question a) and temporal (Question b) impacts upon the PCCBSS' implementation into usual working practices.We have clarified this in the text as follows:“Research sub questions
  a) What organisational contextual factors affect services' implementation (internal impacts)?
  b) How did PCCBSS adapt to delivery and workflow changes imposed by the COVID-19 pandemic (temporal impact)? ”In our analyses, we have used our a priori framework operationalisation (available in Extended data) to cover internal impacts (sub-question A) under Domain 3, Inner setting, and temporal impacts (of which the pandemic is one, sub-question B) as features of Adaptability under Domain 1, Intervention characteristics.
  We have also added text under each of these domains to make this clearer: Intervention characteristics: “Under this domain’s Adaptability construct we will address findings relating to research sub-question b) How did PCCBSS adapt to delivery and workflow changes imposed by the COVID-19 pandemic (temporal impact)?”Inner setting: “Under this domain’s constructs we will address findings relating to research sub-question a) What organizational contextual factors affect services' implementation (internal impacts)?”

We believe we have acted consistently and coherently in our conduct of this research, and hope the clarification in the text against the sub-questions reconciles this reviewer’s uncertainty.

 Reviewer – I would rather suggest to move the main research questions to the end of the introduction and then start with the study design in the methods section. I would move the PPI and ethical approval to the end.

- Author response - Thank you for this suggestion, we agree with moving the research questions to the end of the Introduction and have also moved the PPI and ethics section to the end of the Methods before the Key Outputs section.

Reviewer – Can you provide the interview guide as supplementary material? This would make it easier to "replicate" your methods and use them in other settings.

- Author response - We completely agree it is important to share the topic guide, and had already done so in the Extended data section, but have now made this clearer by detailing its contents:

“Extended data

 OSF: CONNECTED COMMUNITIES | Learning lessons from person-centred community-based support services’ implementation. https://doi.org/10.17605/OSF.IO/TJDP7 (Christian et al., 2023).

 This project contains the following extended data:

 - Operationalisation of CFIR and NPT domains and constructs for Connected Communities .docx

 - Connected Communities WP2 Topic guide V1.2 09 01 2023.docx

 Data are available under the terms of the Creative Commons Attribution 4.0 International license (CC-BY 4.0).”

 However we acknowledge that we had only explicitly indicated within the body text that the operationalisation of our framework constructs was in the OSF project folder (“A full operationalisation of the combined CFIR and NPT implementation coding framework used within this study can be found in the ‘Extended Data’ section below (Christian et al., 2023).”), and that we should also do this for the topic guide too. Thank you for spotting this omission. We have addressed it in the text by inserting the following:

 “The WP2 topic guide questions used in this study with service/linked providers and service users can be found in the ‘Extended data’ section below (Christian et al., 2023).”

 Reviewer – The protocol was published on OSF after the data collection had ended. Please provide a statement on differences between the primary research proposal and the current protocol. Or state that there were none.

- Author response - We did not publish the protocol on OSF, only the framework operationalisation of constructs and the WP2 topic guide questions - but you are correct that these were made available on OSF after completion of data collection. They were made available to support publication of this protocol, rather than having supplementary materials, as the publishing editorial office had advised us to do.

As such, there are no differences between the published protocol and how we conducted the study – there was no other protocol in use. To address this, we have made explicit in the Extended data section what the OSF uploaded documents are:

 This project contains the following extended data:

 - Operationalisation of CFIR and NPT domains and constructs for Connected Communities .docx

 - Connected Communities WP2 Topic guide V1.2 09 01 2023.docx

 Data are available under the terms of the Creative Commons Attribution 4.0 International license (CC-BY 4.0).”

 Reviewer – I am missing information on funding and potential conflicts of interest

- Author response - We’re sorry you were unable to find this information, it has always been available in the PDF format (bottom of p3) and online – this journal’s stylised headings uses the words Competing interests instead of Conflicts of interest, and refers to Grant information rather than Funding:

“Competing interests

 No competing interests were disclosed.

 Grant information

 This project is funded by the National Institute for Health Research (NIHR) under its National Priorities Programme for Adult Social Care and Social Work led by the University of Kent and the Kent Sussex and Surrey ARC (NIHR300099) and awarded to Professor Julien Forder. ‘Connected Communities’ was selected as 1 of the 5 projects to be delivered under the National Priorities Programme, which ARC NWC and University of Central Lancashire are leading in collaboration with the University of Kent and Kent Sussex and Surrey ARC. The views expressed are those of the author(s) and not necessarily those of the NIHR or the Department of Health and Social Care.”

# Reviewer report: Reviewer response for version 1

**DOI:** 10.3310/nihropenres.14643.r30895

**Published:** 2024-01-02

**Authors:** Dragana Vidovic

**Affiliations:** 1 University of Essex, Colchester, England, UK

1. **Is the rationale for, and objectives of, the study clearly described?**

The aim of this project is to better understand how person-centred community-based support services (PCCBSS) are being implemented and embedded into practice and within other services.  The authors correctly identify a lack of understanding of the role and involvement of social care providers in implementing PCCBSS. The current literature and practice lack insights into how PCCBSS is implemented within social care sector as well as across different public services. This work has a potential to significantly contribute to the field of PCCBSS and the overall work of health and social care services.

 With that said, there are aspects of the study that require further clarification. 

 The authors specify that their focus is PCCBSS services who interacted with individuals who self-referred, without providing justification for focusing on self-referral cases in particular (p.5, “Main research question/Aims and objectives”). One the same page 5, authors seems to suggest that they will also include cases where individuals were referred by other services (p.5 “To be eligible, a PCCBSS must have an identifiable operating organisation, and use signposters (variously termed ‘link workers’, ‘social prescribers’, ‘community navigators’, etc.) to support professionally referred and/or self-referred service users to access appropriate individually indicated networks, groups, and resources.”).  The focus should be clarified. 

 Referrals to social prescribing / PCCBSS services come from a variety of sources such as GP, social care, community organisations, family members, self-referral and many others. It is fine to focus on a particular referral pathway; however, it is important to justify why a particular focus on self-referral given the variety of referral pathways. 

 Also, it is not clear if the authors are looking to differentiate between PCCBSS and social prescribing – this would be important to clarify (p. 4, Introduction, 2 ^nd^ paragraph). I would encourage authors to further elaborate on the definition of social prescribing in the most recent literature, ex: https://bmjopen.bmj.com/content/bmjopen/13/7/e070184.full.pdf

 Furthermore, in some sections of the paper authors specify their focus to be on PCCBSS implementation, while in others, the focus is on understanding how PCCBSS can be embedded within the existing frameworks.  In the “Key Outputs” section it is clear that the authors are interested in both aspects and that implementing and embedding of PCCBSS entails specific steps/activities.  However, in the “Main Research Question/Aims and objectives” these two words are being used interchangeably. A PCCBSS project can be implemented at one point in time or across numerous time periods and can be embedded to a varying degree, from minimal interaction with other services to becoming a part of an overall public health strategy in a region.  It is not clear if authors are interested in what organisational contextual factors affect services’ implementation (section “Research sub-questions” question a. refers to implementation), what organisational contextual factors affect services’ embeddedness, or both?  If both, then it needs to be clear that these are distinct processes and not interchangeable (another sub-question needs to be added to include embeddedness). 

 Lastly, in regards to the research question, “b” regarding COVID-19, (How did PCCBSS adapt to delivery and workflow changes imposed by the COVID-19 pandemic?), it is important to make a distinction between a research question and a process of considering background/contextual factors that might impact on the main research question.  Given that PCCBSS were implemented during COVID-19, the impact of the pandemic has to be taken into consideration as the authors suggest on p.4.  However, based on the information provided in the introduction, question “b” cannot be introduced as a research question/sub-question.

 The literature regarding the claim on the impact of social prescribing on the outcomes of interest could be further expanded to provide a more nuanced understanding of the literature in this area.  The National Academy for Social Prescribing webpage provides a detailed overview of the literature across various areas.  The quality of the evidence regarding the impact of social prescribing has been steadily improving over the last 3-4 years and is being continually updated and reviewed to improve ways in which evidence is collected, the type of evidence that is being collected and methods of collection needed for effective project evaluation. 

1. **Is the study design appropriate for the research question?**

Study design is appropriate for the research question/s, however minor edits are needed to ensure that the study reaches it's full potential.  The research question/s should be clarified as mentioned in the response above. 

 Also, given that given that one of the aims of this work is to provide recommendations for setting up future PCCBSS, more information is needed on how three case studies have been selected. Are the three cases representative enough for findings to be generalizable at the wider regional and national level? How were the three cases selected?

 Based on the criteria on p.5, most of the PCCBSS / social prescribing projects would be eligible to be incuded ( “To be eligible, a PCCBSS must have an identifiable operating organisation, and use signposters (variously termed ‘link workers’, ‘social prescribers’, ‘community navigators’, etc.) to support professionally referred and/or self-referred service users to access appropriate individually indicated networks, groups, and resources.”).

 Below, I also provide minor suggestions regarding the Methods section. 

 The “Methods” section starts with Patient and Public Involvement (PPI), with detailed explanation of the PPI framework and it’s importance, yet, it concludes that the public will primarily be involved in only one aspect of the project, namely dissemination. While this is acceptable and understandable, the last sentence of this section comes as a surprise given the previous paragraphs on the importance of PPI framework.  The authors could improve this section by providing a brief explanation of the limited nature of public involvement. 

 In the “Study Design” section where the authors briefly mention CFIR and NPT, it would be useful to explain in 1-2 sentences what these methods are at the outset (“ex. tools used for support implementation and evaluation of interventions in health and social care”).  Theory can mean different things to different readers, and for those readers who might not be familiar with the CFIR or NPT approaches might be left wondering what these refer to.  

1. **Are sufficient details of the methods provided to allow replication by others?**

The authors provide a detailed overview of the methods in the “Combined CFIR and NPT implementation framework” section.  However, for the replication purposes, it would be useful to have a wording of the questions that will be used to conduct semi-structured interviews to discuss pre-determined topics based on the CFIR and NPT (p.6, paragraph 5).  

 Also, what is needed for replication, is a clarity regarding how the case studies have been chosen. 

1. **Are the datasets clearly presented in a useable and accessible format?**

The description of the data collection steps, work packages and coding rules are sufficient to envision that the data will be organized in a useable and accessible format.

Is the study design appropriate for the research question?

Partly

Is the rationale for, and objectives of, the study clearly described?

Partly

Are sufficient details of the methods provided to allow replication by others?

Yes

Are the datasets clearly presented in a useable and accessible format?

Yes

Reviewer Expertise:

Public Health, Social Prescribing, Community Connectedness, Loneliness, Civic Participation

I confirm that I have read this submission and believe that I have an appropriate level of expertise to confirm that it is of an acceptable scientific standard, however I have significant reservations, as outlined above.

# Response

**Published:** 2024-11-05

**Authors:** Danielle Christian

**Affiliations:** University of Central Lancashire, Preston, England, UK

Dear Dr Dragana Vidovic,

 Thank you for taking the time to review our article.

 We really appreciate your recommendations for improvement based on your expertise in this area. We have responded to each of your suggested amendments below.

 Yours sincerely,

 Dr Danielle Christian, on behalf of the authors

**Reviewer** - It is not clear if the authors are looking to differentiate between PCCBSS and social prescribing – this would be important to clarify (p. 4, Introduction, 2 ^nd^ paragraph). I would encourage authors to further elaborate on the definition of social prescribing in the most recent literature, ex: https://bmjopen.bmj.com/content/bmjopen/13/7/e070184.full.pdf

***Author response*** - Thank you for prompting us to clarify this issue in our protocol. Our study is broader than social prescribing alone, which is just one of several forms of person-centred community-based support services that we have explored. We have no objection to citing the newly available conceptual definition of SP from Muhl 2023 (which we have noted in our protocol amendment was published after data collection had been completed in our study. This has replaced the text from the earlier NHS 2020 definition, and Muhl 2023 has been added to the references section (Muhl C, Mulligan K, Bayoumi I, et al. Establishing internationally accepted conceptual and operational definitions of social prescribing through expert consensus: a Delphi study. BMJ Open 2023;13:e070184. doi:10.1136/bmjopen-2022-070184)

 PREVIOUSLY WAS " *The most frequently used term is ‘social prescribing’, which the NHS defines as “local agencies which refer individuals (service users), or permit self-referral, to link worker(s) for engagement with community groups and statutory services who provide personalised practical, emotional, and holistic support to enhance people’s health and wellbeing” (NHS England; NHS England and NHS Improvement 2020).*"

 NOW is " *PCCBSS present in different models and taxonomies, although the most frequently used term is ‘social prescribing’, which is now (published after completion of our data collection) internationally accepted to be conceptually defined as “a means for trusted individuals in clinical and community settings to identify that a person has non-medical, health-related social needs and to subsequently connect them to non-clinical supports and services within the community by co-producing a social prescription — a non-medical prescription, to improve health and well-being and to strengthen community connections” (Muhl 2023).*"

**Reviewer** - Furthermore, in some sections of the paper authors specify their focus to be on PCCBSS implementation, while in others, the focus is on understanding how PCCBSS can be embedded within the existing frameworks.  In the “Key Outputs” section it is clear that the authors are interested in both aspects and that implementing and embedding of PCCBSS entails specific steps/activities.  However, in the “Main Research Question/Aims and objectives” these two words are being used interchangeably. A PCCBSS project can be implemented at one point in time or across numerous time periods and can be embedded to a varying degree, from minimal interaction with other services to becoming a part of an overall public health strategy in a region.  It is not clear if authors are interested in what organisational contextual factors affect services’ implementation (section “Research sub-questions” question a. refers to implementation), what organisational contextual factors affect services’ embeddedness, or both?  If both, then it needs to be clear that these are distinct processes and not interchangeable (another sub-question needs to be added to include embeddedness).

***Author response*** - Thank you for seeking clarity around our use of the term ‘embeddedness’ and its relative use with ‘implementation’.

 As May 2009 states, embeddedness occurs as a result of implementation activity (p540, “Material practices become routinely embedded in social contexts as the result of people working, individually and collectively, to implement them”) – as such they are inherently entangled concepts, with embeddedness being the resulting part of the implementation process (which may happen at a single point in time, or cumulatively from discrete incremental implementation activities), and we have sought to maintain reader clarity by consistently referring to our aim as being around implementation, rather than embeddedness in amending our protocol text.

 Our research sub-questions relate to internal (Question a) and external (Question b) impacts upon the PCCBSS' implementation in to usual working practices, and we have made this clear in the text as follows:

 “ *Research sub questions*

*a) What organisational contextual factors affect services' implementation (internal impacts)?*

*b) How did PCCBSS adapt to delivery and workflow changes imposed by the COVID-19 pandemic (temporal impact)? *”

 We have also added text under each of these domains to make this clearer:

 Intervention characteristics: “ *Under this domain’s Adaptability construct we will address findings relating to research sub-question b) How did PCCBSS adapt to delivery and workflow changes imposed by the COVID-19 pandemic (temporal impact)?*”

 Inner setting: “ *Under this domain’s constructs we will address findings relating to research sub-question a) What organisational contextual factors affect services' implementation (internal impacts)?*”

 We note for transparency though, that we have not amended our framework operationalisation held under the Extended Data section, as we have used these construct definitions in our analyses.

**Reviewer** - The literature regarding the claim on the impact of social prescribing on the outcomes of interest could be further expanded to provide a more nuanced understanding of the literature in this area.  The National Academy for Social Prescribing webpage provides a detailed overview of the literature across various areas.  The quality of the evidence regarding the impact of social prescribing has been steadily improving over the last 3-4 years and is being continually updated and reviewed to improve ways in which evidence is collected, the type of evidence that is being collected and methods of collection needed for effective project evaluation. 

***Author response*** - Thank you for drawing our attention to improving our content in the protocol around recent publications detailing social prescribing evidence quality.

 Coincidentally, the second peer reviewer for the protocol is lead author on a systematic review on social prescribing’s effectiveness as an intervention, which indicates that evidence quality remains low, and that there continues to be an absence of high-quality research. In exploring this and other available literature, we reflected that despite there being an increasing body of evidence for social prescribing, that it is still of variable quality.

 We have included substantially more references to the literature and tightened the statement, which now reads as:  

 “ *The evidence for the effectiveness of social prescribing indicates an absence of high-quality research (Napierala 2022; Khan 2024; Pescheny 2020; Polley 2022; Bickerdike 2017), although studies have reported almost wholly positive impacts from social prescribing (while being limited by a dearth of long-term controlled study availability), with a modest reduction in the use of healthcare resources, and benefits to patients through improvement to their mental and physical health (Dayson et al., 2022; Napierala 2022; Polley 2022; National Academy for Social Prescribing 2022).*”

**Reviewer** - The authors specify that their focus is PCCBSS services who interacted with individuals who self-referred, without providing justification for focusing on self-referral cases in particular (p.5, “Main research question/Aims and objectives”). One the same page 5, authors seems to suggest that they will also include cases where individuals were referred by other services (p.5 “To be eligible, a PCCBSS must have an identifiable operating organisation, and use signposters (variously termed ‘link workers’, ‘social prescribers’, ‘community navigators’, etc.) to support professionally referred and/or self-referred service users to access appropriate individually indicated networks, groups, and resources.”).  The focus should be clarified. Referrals to social prescribing / PCCBSS services come from a variety of sources such as GP, social care, community organisations, family members, self-referral and many others. It is fine to focus on a particular referral pathway; however, it is important to justify why a particular focus on self-referral given the variety of referral pathways. 

***Author response*** - We are interested in all types of referral to PCCBSS (which are broader than social prescribing alone) – the mention of self-referral was to be inclusive of the routes service users take to engage with these services, and is in no way limited to focusing on self-referral alone (hence the use of brackets around ‘self-‘).

 We have clarified this in the text as follows:

*"With regards to ‘Connected Communities’, we are specifically interested in any PCCBSS (across a differentiating range of service intensities, which may (not) include additional support beyond signposting (e.g. models indicated by Husk 2019)) where a signposter consults with a service user, who is (self-)referred, to identify their needs or support to access other local services (regardless of setting, referral route, operating organisation or funding model).“*

**Reviewer** - In regards to the research question, “b” regarding COVID-19, (How did PCCBSS adapt to delivery and workflow changes imposed by the COVID-19 pandemic?), it is important to make a distinction between a research question and a process of considering background/contextual factors that might impact on the main research question.  Given that PCCBSS were implemented during COVID-19, the impact of the pandemic has to be taken into consideration as the authors suggest on p.4.  However, based on the information provided in the introduction, question “b” cannot be introduced as a research question/sub-question.

***Author response*** - We respectfully disagree, as our research sub-questions relate to internal (Question a) and temporal (Question b) impacts upon the PCCBSS' implementation into usual working practices.

 We have clarified this in the text as follows:

*“Research sub questions*

*a) What organisational contextual factors affect services' implementation (internal impacts)?*

*b) How did PCCBSS adapt to delivery and workflow changes imposed by the COVID-19 pandemic (temporal impact)? ”*

 In our analyses, we have used our a priori framework operationalisation (available in Extended data) to cover internal impacts (sub question A) under Domain 3, Inner setting, and temporal impacts (of which the pandemic is one) under Domain 1, Intervention characteristics.

 We have also added text under each of these domains to make this clearer:

 Intervention characteristics: *“Under this domain’s Adaptability construct we will address findings relating to research sub-question b) How did PCCBSS adapt to delivery and workflow changes imposed by the COVID-19 pandemic (temporal impact)?”*

 Inner setting: *“Under this domain’s constructs we will address findings relating to research sub-question a) What organisational contextual factors affect services' implementation (internal impacts)?”*

 We believe we have acted consistently and coherently in our conduct of this research and hope the clarification in the text against the sub-questions reconciles this reviewer’s uncertainty.

**Reviewer** - Given that given that one of the aims of this work is to provide recommendations for setting up future PCCBSS, more information is needed on how three case studies have been selected. Are the three cases representative enough for findings to be generalizable at the wider regional and national level? How were the three cases selected?

***Author response*** - Thank you for seeking clarification on our selection of the case study PCCBSS, we have addressed this in the text, but wish to highlight that we have not claimed the case study services will be generalisable to the wider region or nation.

 Our study extension period permits us to consult other PCCBSS in focus groups to sense-check applicability of our localised regional findings against more diverse populations (e.g. age, ethnicity, rurality) nationally than the North-West Coast’s population allows for, and we will refine our toolkit content based on these discussions, but that is a subsequent piece of work discrete from this study.

 The content now reads as:

 “ *WP1 involves collecting data by reviewing existing service documents from three PCCBSS case studies (identified by convenience snowball sampling of networked member organisations to NIHR ARC NWC, and representative of a local intersecting network of PCCBSS regionally), and WP2 entails interviewing staff and service users (≤20 participants per PCCBSS).*”

**Reviewer** - The “Methods” section starts with Patient and Public Involvement (PPI), with detailed explanation of the PPI framework and it’s importance, yet, it concludes that the public will primarily be involved in only one aspect of the project, namely dissemination. While this is acceptable and understandable, the last sentence of this section comes as a surprise given the previous paragraphs on the importance of PPI framework.  The authors could improve this section by providing a brief explanation of the limited nature of public involvement.

***Author response*** - We can see how the reviewer has come to this assumption and perhaps done ourselves an injustice in the section by not making the 3 levels of PPI actively undertaken clearer.

 We have changed the formatting to support identifying these aspects more readily.

 We have also amended the final sentence to better describe the PPI involvement around coding/analysis interpretation of findings, and local level statement to include review and amendments of public-facing materials – now reads:

 " *The public were not involved in the design and conduct of the study, the choice of outcome measures, or recruitment to the study, but will be integral in reviewing and refining public-facing materials, engaging in initial coding and analysis interpretation discussions, and designing and agreeing plans for dissemination of the study findings and recommendations for moving this work forward.*"

**Reviewer** - In the “Study Design” section where the authors briefly mention CFIR and NPT, it would be useful to explain in 1-2 sentences what these methods are at the outset (“ex. tools used for support implementation and evaluation of interventions in health and social care”).  Theory can mean different things to different readers, and for those readers who might not be familiar with the CFIR or NPT approaches might be left wondering what these refer to.  

***Author response*** - We appreciate this clarification and have addressed this by inserting:

 “ *This study combines two widely-used implementation theoretical tools in a single framework for data collection and analysis to identify and explain key aspects of implementing and embedding new interventions, as also undertaken in earlier implementation work (Burn et al., 2020; Schroeder 2022):*”

**New study added to references:**

 Burn AM, Vainre M, Humphrey A, Howarth E. Evaluating the CYP-IAPT transformation of child and adolescent mental health services in Cambridgeshire, UK: a qualitative implementation study. Implementation Science Communications (2020)1:89. https://doi.org/10.1186/s43058-020-00078-6

 Already referenced:

 Schroeder D, Luig T, Finch TL, Beesoon S, Campbell-Scherer DL. Understanding implementation context and social processes through integrating Normalization Process Theory (NPT) and the Consolidated Framework for Implementation Research (CFIR). Implementation Science Communications (2022)3:13. https://doi.org/10.1186/s43058-022-00264-8

**Reviewer** - The authors provide a detailed overview of the methods in the “Combined CFIR and NPT implementation framework” section.  However, for the replication purposes, it would be useful to have a wording of the questions that will be used to conduct semi-structured interviews to discuss pre-determined topics based on the CFIR and NPT (p.6, paragraph 5).

***Author response*** - We completely agree it is important to share the topic guide, and had already done so in the Extended data section:

*“Extended data*

*OSF: CONNECTED COMMUNITIES | Learning lessons from person-centred community-based support services’ implementation. https://doi.org/10.17605/OSF.IO/TJDP7 (Christian et al., 2023).*

*This project contains the following extended data:*

*- Operationalisation of CFIR and NPT domains and constructs for Connected Communities .docx*

*- Connected Communities WP2 Topic guide V1.2 09 01 2023.docx*

*Data are available under the terms of the Creative Commons Attribution 4.0 International license (CC-BY 4.0).”*

 However, we acknowledge that we had only explicitly indicated within the body text that the operationalisation of our framework constructs was in the OSF project folder *(“A full operationalisation of the combined CFIR and NPT implementation coding framework used within this study can be found in the ‘Extended Data’ section below (Christian et al., 2023).”*), and that we should also do this for the topic guide too. Thank you for spotting this omission. We have addressed it in the text by inserting the following:

*“The WP2 topic guide questions used in this study with service/linked providers and service users can be found in the ‘Extended data’ section below (Christian et al., 2023).”*
